# Ocular manifestations in Gorlin-Goltz syndrome

**DOI:** 10.1186/s13023-019-1190-6

**Published:** 2019-09-18

**Authors:** Antonietta Moramarco, Ehud Himmelblau, Emanuele Miraglia, Fabiana Mallone, Vincenzo Roberti, Federica Franzone, Chiara Iacovino, Sandra Giustini, Alessandro Lambiase

**Affiliations:** 1 Department of Sense Organs, Faculty of Medicine and Odontology, Sapienza University of Rome, Rome, Italy; 2 Department of Internal Medicine and Medical Specialties, Unit of Dermatology, Sapienza University of Rome, Rome, Italy

**Keywords:** Gorlin-Goltz syndrome, Gorlin syndrome, *Nevoid basal cell carcinoma* syndrome, Odontogenic keratocyst, Ocular anomalies, Myelinated optical nerve fiber layers

## Abstract

**Background:**

Gorlin-Goltz syndrome, also known as *nevoid basal cell carcinoma* syndrome, is a rare genetic disorder that is transmitted in an autosomal dominant manner with complete penetrance and variable expressivity. It is caused in 85% of the cases with a known etiology by pathogenic variants in the *PTCH*1 gene, and is characterized by a wide range of developmental abnormalities and a predisposition to multiple neoplasms. The manifestations are multiple and systemic and consist of basal cell carcinomas in various regions, odontogenic keratocistic tumors and skeletal anomalies, to name the most frequent. Despite the scarce medical literature on the topic, ocular involvement in this syndrome is frequent and at the level of various ocular structures. Our study focuses on the visual apparatus and its annexes in subjects with this syndrome, in order to better understand how this syndrome affects the ocular system, and to evaluate with greater accuracy and precision the nature of these manifestations in this group of patients.

**Results:**

Our study confirms the presence of the commonly cited ocular findings in the general literature regarding the syndrome [hypertelorism (45.5%), congenital cataract (18%), nystagmus (9%), colobomas (9%)] and highlights strabismus (63% of the patients), epiretinal membranes (36%) and myelinated optic nerve fiber layers (36%) as the most frequent ophthalmological findings in this group of patients.

**Conclusions:**

The presence of characteristic and frequent ocular signs in the Gorlin- Goltz syndrome could help with the diagnostic process in subjects suspected of having the syndrome who do not yet have a diagnosis. The ophthalmologist has a role as part of a multidisciplinary team in managing these patients. The ophthalmological follow-up that these patients require, can allow, if necessary, a timely therapy that could improve the visual prognosis of such patients.

## Background

The Gorlin-Goltz syndrome (GGS), also termed nevoid basal cell carcinoma syndrome (NBCCS), is a rare condition with estimated prevalence that ranges between 1/30827 and 1/256,000 [1–5]. The disease affects both men and women in rather equal manner [4] and is characterized by a near complete penetrance with variable expressivity [6]. It is inherited in an autosomal dominant manner and is caused in about 50–85% of the cases with a known etiology by pathogenic variants in the tumor suppressor gene *PTCH1* [7], located on chromosome 9 (9q22.3) [1]. In 15–27% of the cases the cause is still unknown [7, 8]. *PTCH1* encodes for a transmembrane receptor which recognizes sonic hedgehog signaling proteins [9]. The Hedgehog cell–cell signaling pathway is crucial for embryogenesis and cell division and its misregulation is implicated in numerous birth defects and cancers. In unstimulated cells, pathway activity is inhibited by the tumor suppressor membrane protein, Patched. Hedgehog signaling is triggered by the secreted Hedgehog ligand, which binds and inhibits Patched, thus setting in motion the downstream events in signal transduction [10–14]. Homozygous inactivation of the *PTCH* gene leads to tumorigenesis and the formation of multiple Basal Cell Carcinomas (BCCs) and other neoplasms [15]. A two-hit model for developmental defects in patients with Gorlin-Goltz syndrome has also been suggested, according to that model subjects inherit one defective copy of the tumor suppressor gene and acquire a “second hit” mutation, such as from ultraviolet light or ionizing radiation [16]. Recently, mutations in suppressor of fused gene (*SUFU*) on chromosome 10q and *PTCH*2 on chromosome 1p have been found in patients meeting criteria for Gorlin-Goltz syndrome [17, 18]. Of note, patients with *SUFU* mutations have an increased risk of developing medulloblastoma as compared to *PTCH1* mutations in Gorlin-Goltz syndrome [6]. De novo mutations represent approximately 20 to 30% of cases [6, 19].

The syndrome has a wide range of manifestations [20–22]. Multiple BCCs are the hallmark feature of Gorlin-Goltz syndrome. Patients can present as early as infancy with BCCs; however, the median age of development is 25 years [23]. the carcinomas may present as classic translucent papules with telangiectasias or may resemble acrochordons (skin tags) [23, 24]. Ovarian and cardiac fibromas (25 and 3% respectively) are also a feature of the syndrome [25].

Major criteria for diagnosis include: multiple (> 2) BCCs or 1 BCC by ≤20 years of age, odontogenic keratocysts of the jaw proven by histology, palmar or plantar pitting, bilamellar calcification of the falx cerebri, bifid/fused/splayed ribs, first-degree relative with NBCCS.

Minor criteria for the diagnosis of the syndrome include: medulloblastoma, increased circumference of the head, congenital malformations (frontal bossing, coarse facies, cleft lip/palate, moderate or severe hypertelorism), other skeletal abnormalities (Sprengel deformity, pectus deformities, syndactyly of the digits), radiologic abnormalities (bridging of the sella turcica, hemivertebrae, fusion or elongation of the vertebral bodies, modeling defects of the hands and feet, or flame-shaped lucencies of the hands or feet), ovarian and cardiac fibromas [20, 23].

Diagnosis of NBCCS requires the presence of two major diagnostic criteria and one minor diagnostic criterion or one major and three minor diagnostic criteria [20, 23], Nonetheless, in most developed countries subjects suspected of having the syndrome are getting genetic testing done in search for PTCH1 mutations as a final confirmation of the diagnosis.

Given that the syndrome has over 100 clinical manifestations and affects many major organ systems, most studies of the Gorlin-Goltz syndrome in the medical literature describe the systemic findings of the syndrome and among those list some ocular manifestations [4, 20].

An article published in 2003 by Graeme C.M. Black et al. studying ocular abnormalities in a series of 30 subjects diagnosed with Gorlin-Goltz syndrome highlighted the vitreoretinal pathologies in this group of patients [26].

Other Articles that deal specifically with the ophthalmological findings are confined to single patient case reports of patients presenting ocular manifestations (hypertelorism, congenital cataract, glaucoma, strabismus, myelinated fibers of the optic nerve, macular pucker, retinal holes, retinal hamartoma and different types of colobomas [4, 15, 24, 25, 27–32]).

This is the first study in which 11 confirmed Gorlin-Goltz patients went through a complete and comprehensive ophthalmologic and orthoptic exams.

## Materials and methods

An observational, cross sectional study was carried out on 11 consecutive patients (7 females and 4 males) with a mean age of 38.5 years (ages range from 18 to 74 years), with previous diagnosis of Gorlin-Goltz syndrome according to the diagnostic criteria of Kimonis (1997), confirmed molecularly with genetic testing, which resulted in 100% of our patients showing a pathogenic variant in the *PTCH1* gene, between May 2017 and July 2018 at the “Sapienza” University of Rome, Italy, in order to assess the involvement of the ocular system in this syndrome”.

All patients went through a complete ophthalmological examination including history, best-corrected visual acuity, intraocular pressure measurement using Goldmann applanation tonometry after topical anesthetic drops application, slit-lamp biomicroscopy, mydriatic indirect fundus biomicroscopy and Spectral domain OCT.

OCT (Optical Coherence Tomography) is a non-invasive, transpupillary and non-contact diagnostic imaging technique that uses the reflection of light signals to obtain a considerable axial resolution of images. It is capable of providing high resolution cross-sections of the retina, optic nerve, vitreous and choroid. Patients were imaged using the Spectral domain OCT (Spectralis Family Acquisition Module, V5.1.3.0; Heidelberg Engineering, Germany) with Heidelberg Eye Explorer (V 1.6.2.0), whose axial resolution was 3.5 μm and the transverse resolution was approximately 15/20 μm, using both the raster scan protocol (20°× 15°, 19 lines of scan) and the radial scan protocol (20°, 6 lines of scan), centered on the fovea. For every single radial protocol scan the presence or absence of vitreoretinal interface pathologies was evaluated to assess the presence of intraretinal and subretinal fluid; in addition some retinal layers integrity, such the external limiting membrane (ELM), the photoreceptor inner segment/outer segment (IS/OS) junction layer and the inner limiting membrane (ILM), was also evaluated.

We made the diagnosis of hypertelorism in accordance with the Tassier classification [33].

When a patient was measured having interorbital distance greater than 30 mm, we consider that patient positive for hypertelorism without further grading of the anomaly.

All patients went through an orthoptical examination including abnormal head positions’ investigation, motor function assessment using the Irvine test, to detect the presence or absence of bifoveal fusion, manifest strabismus as well as the diagnosis of deep amblyopia [34], the cover and uncover test, the convergence test, and corneal reflex evaluation.

We evaluated stereopsis, which is the perception of depth and 3-dimensional structure obtained on the basis of visual information deriving from two eyes, using the Lang test.

Strabismus is defined as a deviation of the primary lines of sight of 1 prism diopter (PD) or more. In strabismus, one eye is either constantly or intermittently not directed toward the same point as the other eye when the patient attempts to fixate an object. As a result, an image of the fixated object is not formed on the fovea of the strabismic eye. The convergent (inward) misalignment of one eye is defined as esotropia; a divergent (outward) misalignment, exotropia; an upward misalignment, hypertropia; a downward misalignment, hypotropia [35].

## Results

Eleven subjects, 7 females and 4 males, with diagnosis of Gorlin-Goltz syndrome were recruited Table 1.

Nine patients (82%) were affected by various degrees of myopia from − 0.5 to − 10 D.

Myopia is an ocular disorder in which the optical power of the eye is too strong for the corresponding axial length. Light rays from an object at infinity entering a non-accommodating myopic eye are converged too strongly and focus in front of the retina. Two patients (18%) showed a high anisometropia, a particular condition characterized by a different refractive power between the eyes, specifically 6 diopters difference in one patient, and 10 diopters difference in the other, two patients (18%) were emmetropes.

Seven patients (63%) presented different types of strabismus with absence of stereopsis: two patients showed esotropia associated with a vertical deviation (V pattern), one patient presented an exotropia associated with vertical deviation (V pattern) and one patient presented only with vertical deviation for inferior oblique overaction. Two other patients were presented with intermittent esophoria/tropia: inward deviation of the eye, usually due to extra-ocular muscle imbalance. The esotropia present in our sample ranged from 6 to 12 prismatic diopters while the exotropia from 10 to 14 prismatic diopters. None of these patients presented diplopia, also known as double vision.

Five patients (45,5%) presented with hypertelorism.

Slit-lamp examination revealed congenital cataract, which is an opacity of the lens present at birth in two patients, (one associated with a reduction in visual acuity (5/10) while the other with a conserved visual acuity). One patient presented with posterior subcapsular cataract in the left eye while another patient presented with bilateral pseudophakia.

One patient was affected by glaucoma with intraocular pressure well controlled by topical pharmacological treatment.

**Fig. 1:**
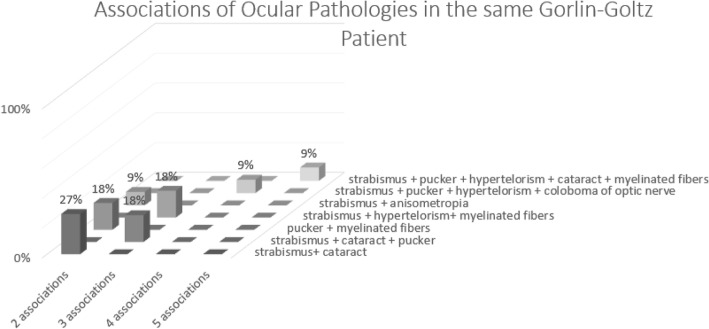
Associations of ocular findings in the same patient

Fundus examination highlighted myelinated optic nerve fiber layers in four of our patients (36%), vitreoretinal interface pathologies in four patients (36%) and coloboma of the optic nerve in one of the patients (9%)(Fig. 1). In particular the vitreoretinal interface pathologies that were observed in four of our patients exhibited different patterns: three eyes presented a retraction of the inner limiting membrane (ILM), a thin and avascular membrane which separates the vitreous body from the retina and plays a role in the pathophysiology of some vitreomacular interface disorders [36], with a preserved visual acuity of 10/10, while two eyes were characterized by a macular pucker (a scar tissue that has formed on the macula and caused wrinkles, creases or bulges to change the flat topography of the macula, necessary for it to function properly), responsible for a reduction in visual acuity (respectively 1/10 and 5/10).

## Discussion

The ocular system has been poorly investigated in Gorlin-Goltz syndrome: our study demonstrates that it is frequently affected and that the main ophthalmological manifestations are myopia, strabismus, myelinated optic nerve fiber layers and hypertelorism. Given that our sample size for the purpose of most relevant statistical analysis falls short, we decided to report only the percentage of patients affected by a certain pathology out of the whole group. Further research in larger groups of patients is needed to determine whether these rates are somewhat accurate.

Myopia is classified into two groups: non-pathologic and pathologic myopia. In non-pathologic myopia the refractive structures of the eye develop within normal limits, however the refractive power of the eye does not correlate with the axial length. The degree of non-pathologic myopia is usually minimal to moderate (< 6.00 diopters) and onset usually begins during childhood or adolescence. Pathologic myopia is classified as a high myopic refractive error that is progressive and presents early in childhood and is defined as spherical equivalent > 6.00 diopters or axial length > 26.5 mm [37]. Patients with high axial myopia are at a greater risk of developing progressive retinal degeneration and other vision threatening pathologies [38]. In our patient population none of the subjects was affected by pathological myopia.

It is important to underline that 73% of our patients presented some pathologies (anisometropia, strabismus, nystagmus) that can cause amblyopia, also known as Lazy eye, which is the loss or lack of development of central vision in one eye that is unrelated to an anatomical problem and is not correctable with lenses. This is consistent with Black et al. series (2003) [26]. It is crucial to detect early these conditions during childhood in order to treat them in time before they can determine amblyopia, for once amblyopia is established, the eye or both eyes involved present a definitive reduction of best-corrected visual acuity.

Other ocular conditions requiring the involvement and follow-up of an ophthalmologist in the management of these patients are vitreoretinal alterations such as epiretinal membranes and macular puckers, for if not detected and surgically treated, they can determine visual impairment and progressive visual loss [39].

Interestingly the patients with GGS showing macular puckers were younger than the average age of subjects diagnosed with a macular pucker in the general population [40]. Another interesting finding of the fundus examination was the frequent presence of myelinated optic nerve fiber layers in these subjects: none of them displayed any visual impairment due to this condition and two of the patients showed both myelinated optic nerve fiber layers and vitreoretinal interface abnormalities. It could be interesting to study the association of these two manifestations in order to understand if they could have a diagnostic value if detected in the same eye or the same patient.

**Fig. 2:**
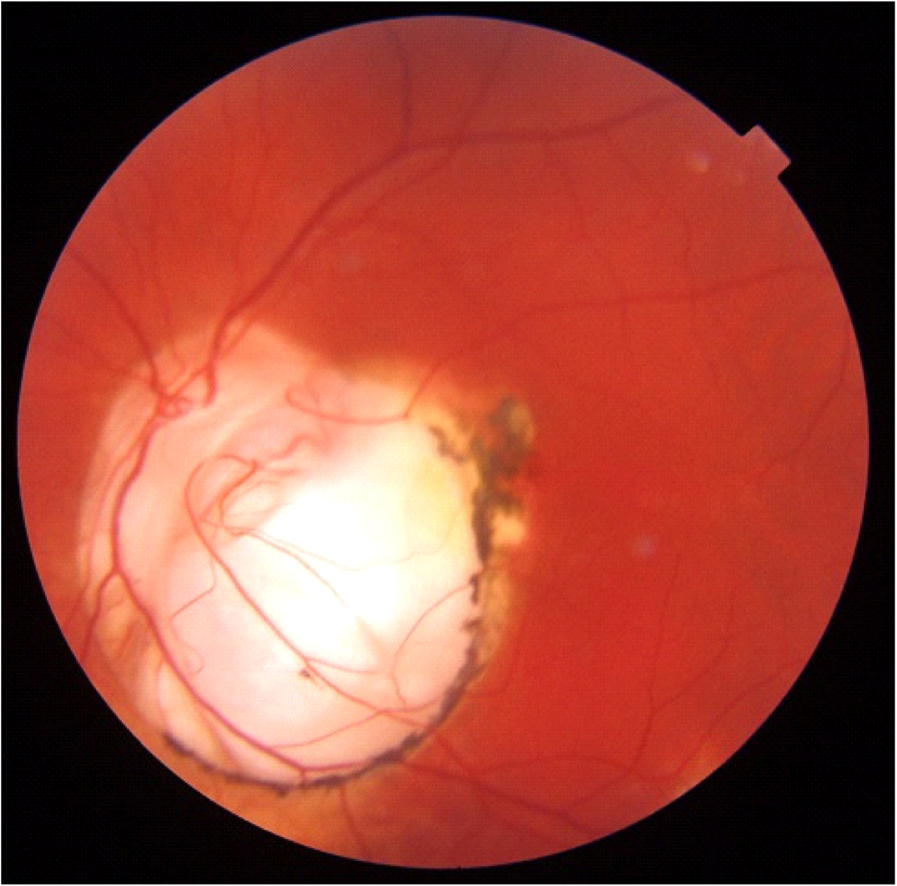
Biomicroscopic photo of one of our patients presenting coloboma of the optic nerve

Associations of ocular pathologies discovered in the same patient (Fig. 2):

**Table 1:** Ocular manifestations with their relative frequencies of occurrence in our patients

Ocular manifestationts	Gorlin-Goltz syndrome
Refractive Errors	
Myopia	9/11 (82%)
Anisometropia	2/11 (18%)
Emmetropia	2/11 (18%)
External examination	
Strabismus	7/11 (63%)
Hypertelorism	5/11 (45,5%)
Nistagmus	1/11 (9%)
Palpebral ptosis	1/11 (9%)
Slit lamp examination	
Cataract/Congenital opacity	2/11 (18%)
Fundus examination	
Myelinated fibers	4/11 (36%)
Epiretinal membranes	4/11 (36%)
Coloboma of the optic nerve	1/11 (9%)

**Fig. 3:**
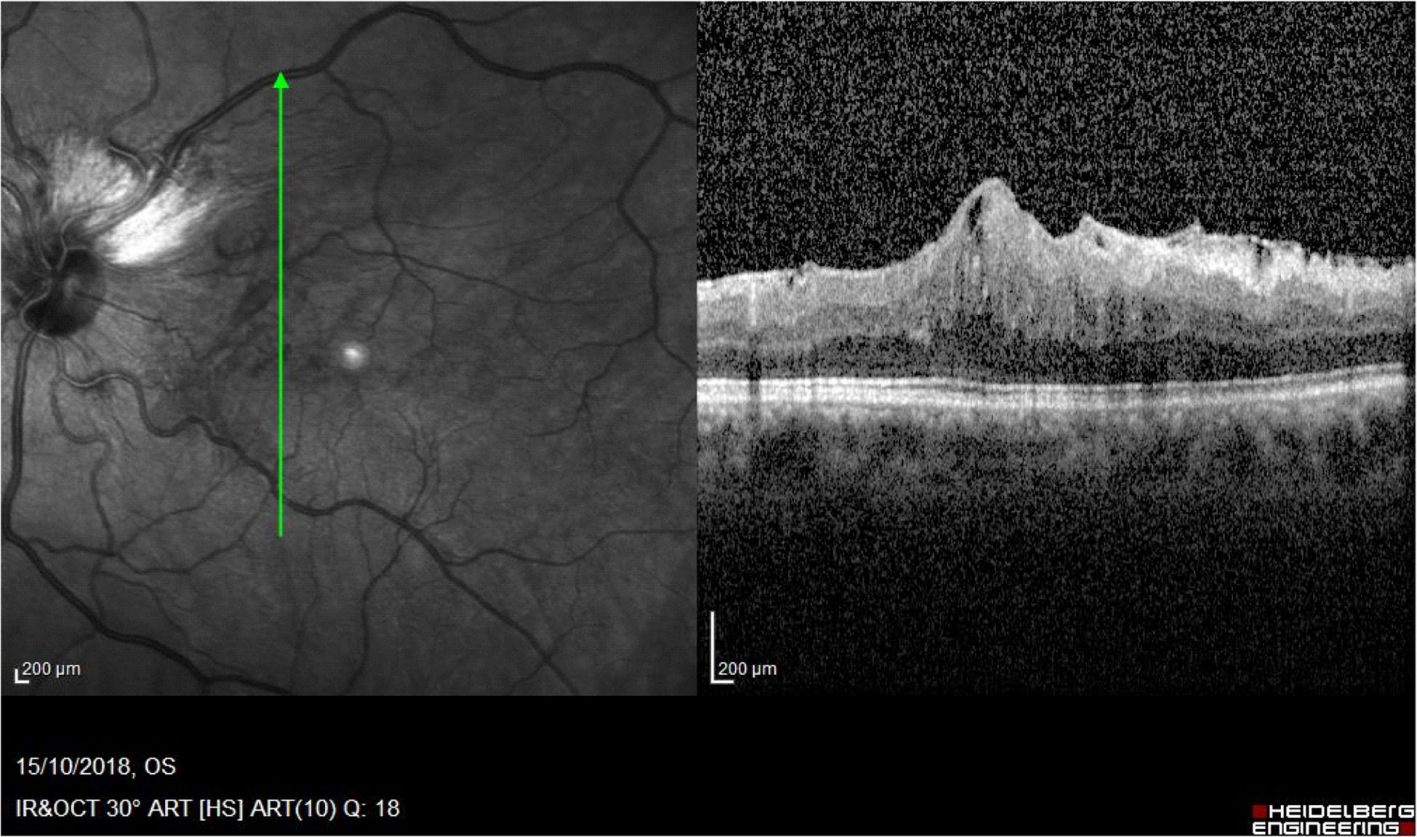
SD-OCT scan showing myelinated optic nerve fiber layers and paramacular pucker in one of our patients with Gorlin syndrome

**Fig. 4:**
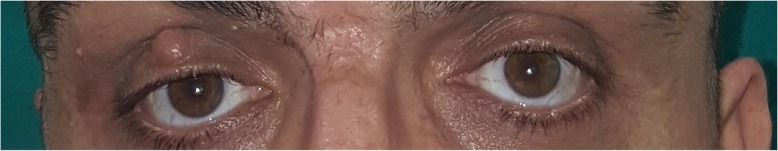
Hypertelorism present in one of our patients

Two associations: Three patients presented with strabismus and cataract. Two patients showed macular pucker and myelinated fibers (Fig. 3). Two patients presented strabismus and anisometropia.

Three associations: Two patients showed strabismus, cataract and macular pucker.

Two patients showed strabismus, hypertelorism and myelinated optic fibers.

Four associations: One patient showed strabismus, macular pucker, hypertelorism. and coloboma of the optic nerve (Fig. 4).

Five associations: One patient presented with strabismus, macular pucker, hypertelorism, cataract and myelinated optic nerve fiber layers.

Coloboma of the optic nerve is a finding that is extremely rare in the general population [41]. *PTCH1* gene plays a key role in embryogenesis, which may explain this finding, although the exact mechanism by which this manifestation occurs is unknown.

## Conclusions

Our data demonstrates that ocular involvement in this syndrome is frequent and tends to concern refractive errors and ocular motility disorders. Some ocular pathologies found in this group of patients, such as macular pucker, coloboma of the optic nerve, congenital cataract and strabismus can cause visual acuity reduction and visual loss. Other manifestations such as hypertelorism and myelinated optic nerve fiber layers can be asymptomatic and do not determine any visual acuity reduction.

Because of the high rate of presentation of the following pathologies in our group of patients, we suggest that the presence of strabismus, myelinated optic nerve fiber layers and/or vitreoretinal interface diseases in the same subject suspected of being affected by the syndrome could increase the suspicion and accelerate the diagnostic process. This is particularly important where genetic testing for this syndrome as a final confirmation of the diagnosis is rarely used.

In conclusion, the study highlights the importance of the ophthalmologist in managing patients with this rare syndrome.

## Data Availability

The datasets generated and/or analysed during the current study are not publicly available due individual privacy concerns but are available from the corresponding author on reasonable request.
